# Cost of incorrect application of antithrombotic prophylaxis prior to invasive procedures

**DOI:** 10.1186/s12913-019-4669-x

**Published:** 2019-11-06

**Authors:** Ma Victoria Cuevas, Ignacio Martínez-Sancho, Jana Arribas, Covadonga García-Díaz, Beatriz Cuevas

**Affiliations:** 1 Hematology Service, University Hospital of Burgos, Avenida Islas Baleares 3, 09006 Burgos, Spain; 2 Primary Care Centre “Gamonal-Antigua”, Av. de los Derechos Humanos.1, 09007 Burgos, Spain; 3 Health Economics Service, University Hospital of Burgos, Avenida Islas Baleares 3, 09006 Burgos, Spain

**Keywords:** Atrial fibrillation, Bridging therapy, Guidelines, Avoidable costs, Workload

## Abstract

**Background:**

We analyze the cost of an incorrect application, by the haematologist, of bridging anticoagulation in patients with low-risk atrial fibrillation (AF) needing interruption of treatment prior to a scheduled invasive procedure. Although not recommended, bridging therapy is widely used, resulting in avoidable costs and increased workload.

**Methods:**

Observational retrospective study. We recorded demographic and clinical data including age, sex, type of procedure, use of bridging therapy with low molecular weight heparin (LMWH), and haemorrhagic complications within 30 days of acenocoumarol withdrawal.

**Results:**

Acenocoumarol was stopped in 161 patients, 97 (60%) were male and 64 (40%) female. Average age was 76,11 ± 8,45 years. Procedures included: minor surgical intervention 58 (36%), colonoscopy 61 (38%), gastroscopy 11 (7%), breast biopsy 4 (2.5%), prostate biopsy 4 (2.5%), infiltration 5 (3%), and other 18 (11%). All patients received bridging anticoagulation with LMWH (40 mg enoxaparin per day) 3 days before and 3 days after the procedure (6 doses). We used a total of 966 doses, at €4.5 per unit, resulted in €4347 of total cost. No complications occurred in 156 patients (97%). Haemorrhage was observed in 5 cases: 1 major haemorrhage needing 6 days of hospital stay and transfusion, and 4 minor haemorrhages (2 patients needed emergency attendance and 2 required hospital admission for 3 and 2 days, respectively). The cost of emergency care was €237.36, and the cost of hospital stay was €6860.81 (€623.71 per day, for 11 days). The total cost of the incorrect application of the protocol was €11,445.17.

**Conclusion:**

Guidelines about bridging anticoagulation in low risk AF patients undergoing scheduled invasive procedures were not followed. This practice increments the complications and supposes an increase in costs besides to an inadequate use of the human resources.

## Background

Anticoagulation therapy with oral vitamin K antagonists (VKA) is indicated for the prevention of thromboembolic events in patients presenting with atrial fibrillation (AF) [1].

Given that embolic and haemorrhagic risks vary among patients, risk stratification scores aimed at identifying candidates for anticoagulation therapy have been proposed [2]. The CHADS_2_ score (Congestive heart failure, Hypertension, Age, Diabetes mellitus, Stroke) is currently considered a simple and reliable tool [3, 4].

In Spain, the oral VKA acenocoumarol is preferentially used [5], mainly because its pharmacokinetics seem to be more predictable and allow easier postoperative management compared to warfarin [6].

However, anticoagulation needs to be stopped prior to any invasive procedure, and approximately 10% of these patients require treatment discontinuation every year [7].

In the perioperative management of these patients, we need to consider the balance between the risk of thromboembolic events and the risk of postoperative haemorrhage.

Douketis et al. [7] reported that AF patients with a CHADS_2_ score of 0 to 2 can be categorized as being at low risk (5% annual risk of thromboembolism after the discontinuation of oral anticoagulation treatment), and bridging anticoagulation is thus not recommended.

Scientific societies have provided guidelines for the interruption and re-initiation of oral anticoagulation within the perioperative period [8]. Depending on the individual risk, anticoagulation needs to be stopped 3 to 5 days before the procedure, and low molecular weight heparin (LMWH) is generally used for bridging anticoagulation.

In recent years, the number of patients receiving anticoagulation therapy has increased with accompanying increases in costs; this is partly attributable to a higher demand from the healthcare workers attending these patients.

Healthcare managers should be aware of the challenges posed by anticoagulation therapy in terms of adherence to the established guidelines, which may help better adjust the workload to the personnel [9].

The objective of this study was to analyse the complications and costs derived from the use of bridging anticoagulation in low-risk AF patients treated with acenocoumarol undergoing invasive procedures.

## Materials and methods

This was a 1-year observational retrospective study that included all patients receiving oral anticoagulation therapy and followed by haematologists belonging to the Anticoagulation Unit of the Haematology Department of the University Hospital in Burgos, in collaboration with general practitioners from several primary care centres within the province of Burgos, Spain.

We included AF patients scoring 0 to 2 on the CHADS_2_ who were treated with acenocoumarol and needed to interrupt the therapy because of a scheduled invasive procedure. Currently, the Anticoagulation Unit of our centre shares responsibility with the primary care physicians and nurses regarding the performance of INR (International Normalized Ratio) tests and the registration of relevant treatment notes in the electronic medical records of these patients.

Our electronic resource TAONET® allows the inclusion of any relevant information inside the “Comments” section, such as the need for anticoagulant therapy discontinuation prior to surgery or other invasive procedures. Additionally, the haematologist can prescribe and register the dose of acenocoumarol, specify when to withdraw the treatment and recommend the best way of substituting another anticoagulant.

We searched for haemorrhagic complications recorded in the patients’ clinical records that occurred within 30 days of the interruption of oral anticoagulant therapy due to an invasive procedure.

Patient demographic and clinical data, the reasons for therapy discontinuation, the type of invasive procedure, the use of bridging anticoagulation, and the types of haemorrhagic complications were recorded and analysed. Laboratory data, including haemoglobin values and the number of units of packed red blood cells transfused, were also recorded.

We analysed each occurrence of haemorrhagic complications in terms of emergency unit assessment, the need for hospital admission, the need for transfusion, hospital stay attributable to haemorrhagic complications, and the final outcome.

A major haemorrhagic event was defined as a lethal haemorrhage, symptomatic haemorrhage in a critical organ (intracranial, intra-spinal, intra-ocular, retroperitoneal, intra-articular, pericardial, and intramuscular provoking a compartment syndrome), haemoglobin value decrease of more than 20 g/L or the need for the transfusion of 2 or more units of packed red blood cells [10]. Non-life-threatening haemorrhages (epistaxis, ecchymosis or haematuria) not needing transfusion were considered minor haemorrhagic events.

All clinical data were included in a Microsoft Office Excel 2010 spreadsheet and later exported and analysed with IBM SPSS v.19 software. A descriptive statistical analysis was performed that included the costs of bridging therapy and haemorrhagic complications attributed to LMWH treatment.

## Results

Within the study period, oral acenocoumarol treatment was interrupted prior to a scheduled invasive procedure in 161 AF patients scoring 0 to 2 on CHADS_2_. The average age was 76.1 years (standard deviation, 8.5 years); 64 (40%) were female (average age 77.0 ± 8, 09), and 97 were male (average age 75.5 ± 8.66). Table 1 shows the types of procedures performed.

**Table 1:** Invasive procedures

Procedure	Patients (n)	Patients (%)
	161	100%
Minor surgical intervention	58	36
Colonoscopy	61	38
Gastroscopy	11	7
Prostate biopsy	4	2,5
Breast biopsy	4	2,5
Infiltration^a^	5	3
Other procedure^b^	18	11

^a^Knee, left saphenous neuropathy, internal anal sphincter, ankle and left femorocutaneous nerve

^b^salivary gland biopsy, implant of intraocular corticoid delivery device, cystoscopy (5 patients), biopsy of cavum, lung biopsy, thyroid nodule biopsy (3 patients), bronchoscopy, lumbar puncture, endometrial biopsy, pancreatic mass biopsy, renal mass biopsy and sentinel node biopsy

No complications occurred in 156 patients (97%). Five patients (3%) had haemorrhagic complications: 1 had a major haemorrhage, and 4 had minor haemorrhages (Table 2).

**Table 2:** Patients’ clinical features

Variable	Female (n/%)	Male (n/%)	Global (n/%)
Sex	64 (40)	97 (60)	161 (100)
Age (years)	77. 03 (± 8, 09)	75.51 (± 8,66)	76.11 (± 8.45)
Invasive procedure			
Minor surgical intervention	23 (39.6)	35 (60.4)	58 (36)
Colonoscopy	24 (39.3)	37 (60.7)	61 (38)
Gastroscopy	6 (54.5)	5 (45.5)	11 (7)
Prostate biopsy		4 (2.5)	4 (2.5)
Breast biopsy	4 (2.5)		4 (2.5)
Infiltration	0	5 (3)	5 (3)
Other procedures	7 (38.8)	11 (61.2)	18 (11)
No complications	62 (38.5)	93 (58.5)	156 (96.9)
Complications:	2	3	5 (3.1)
Major haemorrhage	1 (0.6)	0	1 (0.6)
Minor haemorrhage	1 (0.6)	3 (1.8)	4 (2.4)

All patients recovered from the haemorrhagic complication without sequelae. All patients received 40 mg per day subcutaneous enoxaparin for 6 days; acenocoumarol was stopped 3 days before the scheduled procedure, and it was re-initiated the same day as the procedure.

Bridging anticoagulation was begun 3 days prior to the procedure and maintained for 3 days afterwards, thus overlapping with acenocoumarol.

We used 966 doses of enoxaparin for the entire cohort, with a cost of €4.50 per dose, yielding a total cost of €4347.

The cost of attendance at the Emergency Unit was €237.36 (€118.68 per patient), and the cost related to hospital admissions was €6860.81 (€623.71 per day, 11 days). Thus, we estimate the total cost of an incorrect application of antithrombotic prophylaxis to be €11,445.17 (Table 3).

**Table 3:** Cost of incorrect application of antithrombotic prophylaxis

	Use of LMWH and healthcare resources	Euro	Total (€)
Bridging anticoagulation (LMWH)	966 (6 doses/161 patients)	4.5 €/unit	4.347
Emergency Unit	2 patients	118.68 €/ patient	237.36
Hospital Admission	3 patients (11 days)	623.71 €/day	6860.81
Total cost			11,445.17

## Discussion

According to the recommendations provided by the American College of Chest Physicians (ACCP 2012), the thrombotic risk in patients previously receiving treatment with oral anticoagulants who need treatment interruption because of an invasive procedure, is classified into three groups [7]. Patients presenting with AF and a CHADS_2_ score of 0 to 2 (without a history of stroke or transient ischaemic event) and patients with a history of venous thromboembolism at least 12 months before but no other risk factors belong to the low risk group. However, evidence about the perioperative management of oral VKA is limited and stems from observational studies and recommendations provided by scientific societies [11]. In fact, there seems to be a great discrepancy between the opinions of haematologists and primary care physicians regarding the timing and appropriateness of bridging anticoagulation prior to invasive procedures.

To clarify this controversy, Douketis et al. conducted the BRIDGE study, a double-blind, placebo-controlled randomized trial in which, following the interruption of warfarin therapy prior to an invasive procedure, patients were randomized to receive either bridging anticoagulation with LMWH or a placebo. The objective was to identify the need for bridging anticoagulation in AF patients undergoing invasive procedures. The primary endpoints of the study included the occurrence of arterial thromboembolism and major bleeding episodes within 30 days after the procedure.

The study included 1884 patients: 950 received the placebo, and 934 received bridging anticoagulation. The incidence of thromboembolism was 0.4 and 0.3% in the placebo and treatment groups, respectively, with a risk difference of 0.1 percentage points (95% confidence interval [CI], − 0.6 to 0.8; *P* = 0.01 for noninferiority). However, the rates of major haemorrhagic episodes were 1.3 and 3.2% in the placebo and treatment groups, respectively (relative risk, 0.41; 95% CI, 0.20 to 0.78; *P* = 0.005 for superiority). The authors concluded that patients who received bridging therapy had not only similar rates of thromboembolic complications but also fewer major bleeding episodes [12].

In our study, in which bridging anticoagulation was used in all patients, we observed incidence rates of 0.62 and 2.4% for major and minor bleeding events, respectively.

The misuse of bridging anticoagulation by haematologists and primary care physicians is a matter of concern among scientific societies, and these societies have questioned this practice [13]. Moreover, the Canadian Haematology Society has suggested that bridging anticoagulation should not be offered to patients unless the risk of thrombosis clearly outweighs the risk of haemorrhage, given that the majority of patients will likely not benefit and that it might result in unwanted complications, increasing the cost and avoidable work overload [14].

The study by Rios et al. has shown that, in AF patients receiving VKA treatment, bridging anticoagulation prior to an invasive procedure was independently associated with a higher risk of all peri-procedure complications [15].

In Spain, the use of warfarin is relatively low (5.2%), and we lack specific data regarding bridging anticoagulation. However, the management of non-valvular AF patients receiving acenocoumarol does not seem to be different from the management of patients receiving warfarin, and data are generalizable [16].

An increasing number of patients receiving VKA necessarily means more personnel working in Anticoagulation Units. In our area, the management and control of oral anticoagulation therapy has been decentralized and shared with general practitioners using a telemedicine system.

This system enhances communication between haematologists and primary care physicians, reduces costs attributed to specialized consultations, and improves patient satisfaction [17, 18]. This decentralization process granted new competencies to primary care physicians, who have demonstrated good quality management of this therapy [19, 20]. In our centre, the use of electronic clinical records allows us to issue recommendations about the withdrawal of anticoagulation, so the patient can undergo bridging anticoagulation as an outpatient treatment, thus avoiding a hospital stay, as also reported by Pappas et al. [21]. However, costs do increase whenever anticoagulation withdrawal and bridging therapy are unnecessarily offered to patients. Given that the cost of controlling oral anticoagulation therapy is lower in primary care than in specialized consultation [22], it seems reasonable that recommendations about anticoagulation withdrawal prior to invasive procedures should ideally be issued by primary care physicians, thus reducing specialist workload. We believe that primary care physicians should be involved in the implementation of oral anticoagulation guidelines. A close observation of such guidelines should result not only in fewer adverse effects and complications but also in reduced costs [23].

In our centre, we have been using unnecessary bridging therapy with LMWH, which has resulted in cases of post-procedure haemorrhage. This practice has increased costs because of emergency attendance and hospital stays for the management of haemorrhagic complications. In Europe, such a discrepancy between the recommendations in the guidelines and clinical practice has led to the design of electronic resources aimed to help with the correct application of protocols and to enhance adherence to the guidelines [24].

## Conclusions

An incorrect application of the guidelines may increase the risk of haemorrhage, so efforts should be made to update local procedures according to international recommendations. Subsequently, the implementation of protocols based on such recommendations requires periodic analysis and follow-up to ensure correct adherence and better control and rationalization of costs and personnel workload.

## Data Availability

All data generated or analysed during this study are included in this published article. The datasets used and/or analysed during the current study are available from the corresponding author on reasonable request.

## Abbreviations

ACCP: American College of Chest Physicians
AF: Atrial fibrillation
CEIm: Drug Research Ethics Committee
CHADS2: Congestive heart failure, Hypertension, Age, Diabetes mellitus, Stroke
INR: International Normalized Ratio
LMWH: Low molecular weight heparin
VKA: Vitamin K antagonists
